# Analysis of expression profiles of selected genes associated with the regenerative property and the receptivity to gene transfer during somatic embryogenesis in *Triticum aestivum* L.

**DOI:** 10.1007/s11033-013-2696-y

**Published:** 2013-09-29

**Authors:** Fabienne Delporte, Yordan Muhovski, Anna Pretova, Bernard Watillon

**Affiliations:** 1 Department of Life Sciences, Bioengineering Unit, Walloon Agricultural Research Centre (CRAw), Chaussée de Charleroi 234, 5030 Gembloux, Belgium; 2 Institute of Plant Genetics and Biotechnology, Slovak Academy of Sciences, Akademicka 2, P.O. Box 39 A, 950 07 Nitra, Slovakia

**Keywords:** Developmental window, In vitro tissue culture, Genetic transformation, Mature embryo, Regeneration, Somatic-to-embryogenic transition

## Abstract

The physiological, biochemical and molecular mechanisms regulating the initiation of a regenerative pathway remain partially unknown. Efforts to identify the biological features that confer transformation ability, or the tendency of some cells to induce transgene silencing, would help to improve plant genetic engineering. The objective of our study was to monitor the evolution of plant cell competencies in relation to both in vitro tissue culture regeneration and the genetic transformation properties. We used a simple wheat regeneration procedure as an experimental model for studying the regenerative capacity of plant cells and their receptivity to direct gene transfer over the successive steps of the regenerative pathway. Target gene profiling studies and biochemical assays were conducted to follow some of the mechanisms triggered during the somatic-to-embryogenic transition (i.e. dedifferentiation, cell division activation, redifferentiation) and affecting the accessibility of plant cells to receive and stably express the exogenous DNA introduced by bombardment. Our results seem to indicate that the control of cell-cycle (S-phase) and host defense strategies can be crucial determinants of genetic transformation efficiency. The results from studies conducted at macro-, micro- and molecular scales are then integrated into a holistic approach that addresses the question of tissue culture and transgenesis competencies more broadly. Through this multilevel analysis we try to establish functional links between both regenerative capacity and transformation receptiveness, and thereby to provide a more global and integrated vision of both processes, at the core of defense/adaptive mechanisms and survival, between undifferentiated cell proliferation and organization.

**Electronic supplementary material:**

The online version of this article (doi:10.1007/s11033-013-2696-y) contains supplementary material, which is available to authorized users.

## Introduction

For in vitro culture-based plant transformation methods, amenability to the culture stages required for gene transfer, selection and plant regeneration is a major determinant of transformation efficiency, which is controlled by intrinsic and external factors.

Although considerable progress has been made in the past decade, the ability to transform Triticeae species currently still lags behind that of other model plants, such as *Arabidopsis thaliana* or rice (*Oryza sativa* L.). Within Triticeae, while stable transformation is relatively efficient in barley (*Hordeum vulgare* L.), for the other crops, particularly wheat (*Triticum aestivum* L.), transformation efficiency continues to improve, but the establishment of a high throughput transformation platform and the strong genotype dependence of transformation methods, among others, remain technical hurdles [1–4].

The delivery of DNA into cells competent for in vitro culture regeneration is one of the crucial requirements for achieving genetic engineering of plant cells. Monocotyledonous species have long been described as recalcitrant in terms of in vitro regeneration. Compared with the two other major cereals, rice and maize (*Zea mays* L.), wheat appeared even more refractory [5]. Embryogenic cultures remain the reliable and main route in regenerating transgenic cereals, regardless of the DNA transfer strategy. Considered as the most responsive explants, immature zygotic embryos (and derived calli) are the usual target tissues for wheat genetic transformation, through either microprojectile or *Agrobacterium*-mediated DNA delivery [5, 6].

There is growing interest in developing transformation protocols and methods using mature seeds rather than the immature embryos as the starting explants in developing high throughput transformation pipelines, such as those developed for rice. The use of mature embryos as starting material provides a low-cost and time-saving alternative to explants from immature plants [4]. In rice, *Agrobacterium*-mediated transformation using mature embryo-derived calli is becoming the method of choice for most laboratories, allowing up to several thousand independent lines to be generated for large-scale applications [7, 8].

We had previously developed a simple and effective procedure for wheat, based on mature embryo in vitro culture and biolistic DNA delivery [9, 10]. This method provides an appropriate experimental model for studying the regenerative capacity of plant cells through somatic embryogenesis and their receptivity to exogenous DNA [11].

Only a subset of cells are competent for regeneration but those cells are not necessarily transformable and not every transformed cell can be regenerated into plants [12]. Our previous results demonstrated varying abilities in transient and stable marker gene expression between callus cells derived from an immature zygotic embryo and those derived from a mature one. Whether the recipient tissue variations mentioned in this report [9] could be attributed to their proliferative potential, physiological fate or the possible existence of different dynamics during tissue culture was an interesting issue to address.

In addition, in our model, among several culture periods tested the most suitable physiological state for achieving stable transformation was obtained with a 6 day-pre-culture period [9]. This pivotal period in terms of receptivity to exogenous DNA transfer coincides with a critical stage of the multi-step regenerative pathway. At this time the tissue cultures go through a transition phase between unorganized cell proliferation and differentiation [10]. The developmental switching of somatic cells towards the regenerative pathway involves a plethora of adjustments in response to the in vitro environmental conditions (i.e., a whole reorganization of their physiology, metabolism and morphology), dedifferentiation and the acquisition of a ‘stem cell’-like state that confers pluripotency, requiring remodeling of the gene expression program [13–15].

Our intention in this study was to improve the understanding of the functional links between the regenerative capacity and the transformation receptiveness. On the premise that cells are dynamic systems that go through a sequence of physiological stages in time and that these physiological events might interfere with their transformation receptiveness, we investigated molecular events underlying the responses to in vitro culture and bombardment transformation in tissues proliferating from mature wheat embryos.

Transcriptional profiling has shown that many genes are differentially expressed during somatic embryogenesis (SE) induction or differentiation in cereals. The genome-wide shift in the transcriptome involves multiple cellular pathways interconnecting within an intricate functional network; these pathways include signal transduction cascades, defense, anti-oxidation, programmed cell death/senescence, hormone response/metabolism and cell division [16, 17].

Choosing a candidate-gene strategy, semi-quantitative reverse transcription and polymerase chain reaction (RT-PCR) studies were conducted to follow the expression of genes whose functions were associated with key events or steps in both the expression of totipotency in vitro and the transformation mechanisms. We characterized the transcription profiles for genes for which expression data might provide spatio-temporal indicators relative to: (1) the establishment of plant defense/adaptive mechanisms and antioxidant apparatus dynamics; (2) cell adjustment to changing environmental conditions and reprogramming of genome expression; and (3) cell division activation and a proliferative state, competent when it comes to the expression of totipotency, as well as for gene transfer and transgene integration [11].

Genes investigated experimentally are associated with: (1) response to stress (glutathione *S*-transferase, GST), (2) transcriptional regulation (MADS-domain transcription factor, MADS-box), (3) cell proliferation and S phase-enriched cell populations (replication factor complex, RFC, known to be associated with S phase, i.e. DNA synthesis phase of the cell cycle) and (4) acquisition/maintenance of embryogenic competence (somatic embryogenesis receptor kinase, SERK). In addition, the involvement of germin oxalate oxidases (GerOXOs) in stress response, developmental reprogramming and differentiation (including through SE and microsporogenesis), reactive oxygen species (ROS) (H_2_O_2_) and calcium release, cell wall remodeling and reinforcement, and defense strategy in Poaceae makes this family particular attractive [18–22]. This gene family might provide an appropriate spatio-temporal molecular signature of the successive stages of both competencies in cereals.

The results from the transcript profilings and enzymatic oxalate oxidase (OXO) activity are discussed in relation to tissue culture response and transformation receptiveness. Finally, macroscopic, microscopic and molecular data were summarized from a holistic perspective and integrated with experimental data and theoretical aspects in the literature in order to address the question of tissue culture and transgenesis more broadly.

Any attempt to learn more about the events triggered during the somatic-to-embryogenic transition would help to increase understanding of totipotency and plant ontogenesis flexibility. Understanding the biological features that confer transformation ability, or the differences in the tendency of some cells to bring about transgene silencing, is desirable for a rational approach of plant genetic engineering. From a practical perspective in wheat transformation, molecular and biochemical signatures of the main steps in the expression of totipotency and receptivity to transgenesis would be useful tools, whatever the regenerative pathway or the gene transfer method.

## Materials and methods

### Plant material

The donor plants (*T. aestivum* L.) of the winter cultivar Dream were grown in the field. Harvested mature seeds were stored at room temperature.

### Seed surface-sterilization and aseptic embryo isolation

Wheat caryopses were fungicide treated (Sibutol ^®^ Bayer) against seed-borne pathogenic fungi at least 8 days before culture initiation, after which seeds were kept in a flask for several weeks for subsequent use. After a 16-h rehydration in sterile water at room temperature, the seeds were surface-sterilized with 70 % ethanol for 2 min, soaked in 8 % calcium hypochlorite containing 0.1 % Tween 80 (Merck) for 10 min and rinsed three times with sterile deionized water. The embryos were aseptically removed and protected from desiccation.

### Tissue culture

Thin tissue fragments (≈500 fragments resulting from crushing 100 embryos) were re-suspended in 4 ml of liquid basal medium (i.e., MS medium, supplemented with 100 mg/l casein hydrolysate [Sigma] and 20 g/l sucrose [Merck] as the carbon source, pH adjusted to 5.8 with NaOH prior to autoclaving). The resulting suspension was distributed among five 9-cm disposable plastic Petri dishes containing the callus induction medium (i.e., the liquid basal medium containing 2 mg/l filter-sterilized 2,4-D (Sigma) as the growth regulator, semi-solidified with 7 % agar [Invitrogen]. Excess liquid medium was retrieved and discarded before sealing the dishes. Light and temperature conditions for callus culture and plant regeneration were as described earlier [9].

### Molecular analysis

#### Sampling

Nine specimens were used for the RT-PCR experiments. The sampling consisted of five culture periods (2, 6, 8, 14 and 20 days) and two morphogenetic types (embryogenic and non-embryogenic that were distinguishable after a 6-day period of tissue culture).

#### Total RNA extraction

RNA was extracted in at least three independent replications. The tissues (*T. aestivum* L. Dream cv) (500 mg) were frozen in liquid nitrogen and disrupted using a mortar and pestle. Total RNA was extracted (‘Pure Script RNA isolation Kit^®^’ GENTRA SYSTEMS for GST and MADS-box analysis, ‘TRI REAGENT^®^’ FERMENTAS for RFc, SERK and GerOXO analysis) and treated with DNase I (‘Dnase I RNase free^®^’ ROCHE) to remove any residual genomic DNA, according to the manufacturer’s instructions. RNA yield was quantified by spectrophotometry and RNA integrity was controlled by separation on a 1.2 % agarose gel electrophoresis, each sample containing 0.5 mg/ml ethidium bromide. The RNA quantity, quality and integrity were assessed by agarose gel electrophoresis and by evaluating the 28S and 18S ribosomal RNA bands.

#### Semi-quantitative one-step RT-PCR

Sequence specific primers were designed for each gene as following. For 18SrRNA, GST and RFC, primers previously used in cited papers (Table 1) were selected for our study. For MADS-box, SERK and GerOXO, primers were designed from published sequences (Table 1) using Primer Premier Software (version 5.0). In all cases, a single amplicon of the expected size was detected.

**Table 1:** Information on the primers used for RT-PCR

Acronym	Origin definition	Gene bank accession number	Primer sequences	Amplicon size (bp)
*18S ribosomal RNA*				
18S rRNA	*Triticum aestivum* 18S rRNA gene for 18S ribosomal RNA	AJ272181	Forward 5′-atgataactcgacggatcgc-3′ Reverse 5′-cttggatgtggtagccgttt-3′ [202]	169
*Glutathione-S-transferase*				
TtGSTU1	*Aegilops tauschii* glutathione-*S*-transferase 2 and glutathione *S*-transferase 1 genes, complete cds	AY013753	Forward 5′-aagggcctgagctacgag-3′ Reverse 5′-tgctggcggctcacttg-3′	622
TtGSTU2	*Aegilops tauschii* glutathione S-transferase gene, complete cds	AY013754	Reverse 5′-gtgtgctggctcagttag-3′	622
TtGSTU3			Reverse 5′-gcatcaagcgagccgaaac-3′ [25]	527
*Replication factor*				
Rfc-1	*Triticum* sp. partial mRNA for replication factor C, large subunit	AJ318783	Forward 5′-aatgcaagtgatagtcgtggtaaag-3′ Reverse 5′-cctatccaaattagcttgctgtgat-3′ (adapted from [27])	990
*Somatic embryogenesis receptor kinase*				
TaSERK1	*Triticum aestivum* mRNA clone, complete cds	BT009426	Forward 5′-gaactccaattccaaacagaag-3′ Reverse 5′-cattagccatgtatggata-3′	125
*MADS-domain transcription factor*				
MADS-box	*Triticum aestivum* mRNA clone, partial cds, putative homologous to *TaMADS#12* mRNA for MADS box transcription factor	AB007505	Forward 5′-ccttctccaagcgccgcaac-3′ Reverse 5′-gaactcgtagagcttgccgc-3′	113
*Germin oxalate oxidase*				
Ger oxo	*Triticum aestivum* germin protein precursor, mRNA, complete cds	M21962	Forward 5′-agatcggcatcgtgatgaaaggt-3′ Reverse 5′-gggttctggctgttgaaggagac-3′	190

Full reference gene names, primer sequences and amplicon sizes

Reverse transcription and cDNA amplification were performed in a single tube experiment using ‘Ready-To-Go™ RT-PCR Beads’ (GE HEALTHCARE, formerly Amersham Biosciences), according to the instruction manual protocol.

Briefly, first strand cDNAs was synthesized from 200 μg of total RNA using an oligo dT as the first-strand primer and the Moloney murine leukemia virus reverse transcriptase at 42 °C for 20 min. The enzyme and RNA:cDNA heteroduplex were denatured at 95 °C for 5 min. Sequence specific primers were then added to the reaction mixtures for the selective amplification of the cDNAs used as templates (Table 1). The PCR reactions were performed under the following conditions: 1 min denaturation at 95 °C, 1 min annealing, 2 min polymerization at 72 °C and a 10 min end-elongation step at 72 °C. Specific conditions (annealing temperature-cycle numbers) were 56 °C-30, 60 °C-35, 58 °C-35, 58 °C-35, 56 °C-35 and 56 °C-30, for 18SrRNA, GST, MADS-box, RFC, SERK and GerOXO analysis, respectively.

The amplified product sizes were confirmed by separation on a 1.2 % agarose gel electrophoresis. Ethidium bromide-stained signals were digitalized and quantified by the Quantity One^®^ 4.2.1software package (BIORAD Laboratories) and expressed as total pixel values. The amount of transcripts was normalized to the amount of the housekeeping transcript, 18sRNA (i.e., normalized results were determined as ratios of the total pixel value of the band of interest to the total pixel value of the 18S rRNA band). For each transcript, the final relative abundance was expressed as a percentage of the highest value among the set of observations (i.e., nine samples: 2, 6 days embryogenic, 6 days non-embryogenic, 8 days embryogenic, 8 days non-embryogenic, 14 days embryogenic, 14 days non-embryogenic, 20 days embryogenic and 20 days non-embryogenic). All the molecular analyses were performed at least three times.

### Statistical analysis

Calculated variables were normalized (arcsinrac[x] variable transformation) and the data were analyzed by analysis of variance (ANOVA). Mean differences were compared pair-wise with the Tukey Multiple comparison procedure, at a confidence interval of 95 % (Systat for Windows, Version 8, SYSTAT Software Inc.).

### Histochemical detection of OXO activity

Oxalate oxidase activity in situ was detected as described by Caliskan and Cuming [23] using the procedure reported by Dumas et al. [24]. The tissues were incubated with an assay buffer (25 mM succinic acid, 3.5 mM EDTA, 2.5 mM oxalic acid, 0.6 mg/ml 4-chloro-1-naphthol, pH 4.0) at 25 °C in night darkness, and were fixed in 4 % paraformaldehyde in phosphate buffered saline (PBS). The control samples were incubated in assay buffer lacking oxalic acid.

## Results

**Fig. 1:**
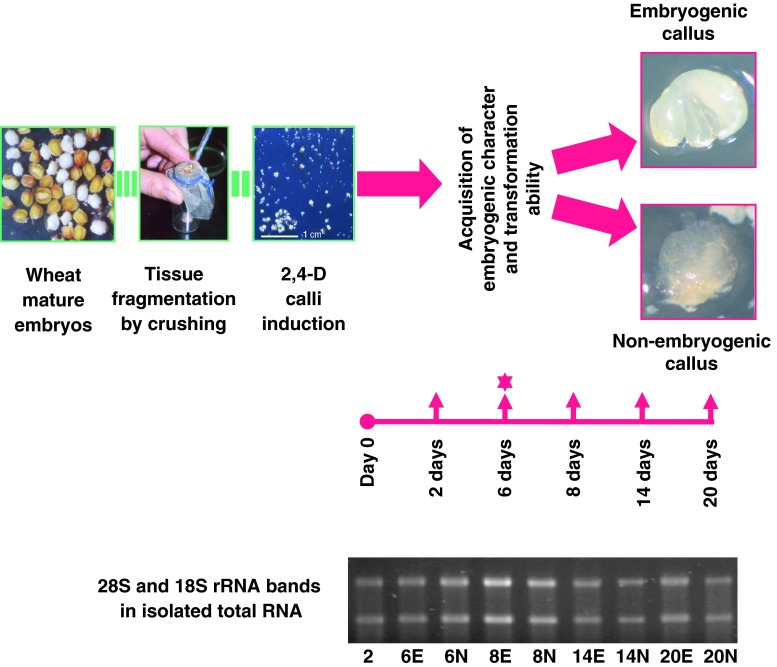
Wheat mature embryo culture, sampling for RNA extraction and relative quantification of target gene transcripts. Samples were taken at regular periods after calli induction (*red arrows*, pointing *upwards*) from tissues displaying distinct behavior (i.e., embryogenic vs. non-embryogenic, and lesser vs. optimal ability for long-term transformation). The embryogenic character becomes apparent after 6 days of culture; this period also coincides with the most suitable physiological state for bombardment DNA introduction and long-term transgene expression in calli [9, 10]. This 6 day-culture period corresponds to a pivotal developmental window. The two types of calli with distinct morphogenetic capacities are illustrated in this figure (on the *right*, 7-day-old calli). Calli termed ‘embryogenic’ (*above*) are *white* to *greenish-white* and compact, with smooth-looking structures formed by densely proliferating cells. These calli tend to engage in the SE differentiation pathway, subsequently confirmed by cell cluster greening (i.e., chloroplast differentiation) and followed by the emergence of shoots (i.e., somatic embryo germination). The calli considered to be ‘non-embryogenic’ (*below*) are *white*, limpid, watery, friable and formed by large highly vacuolated cells. Using semi-quantitative RT-PCR and gel analysis, the 18S ribosomal RNA gene was used as the control for normalization and relative quantification of target gene RNA abundance. *2*, *6*, *8*, *14*, and *20* number of days after 2,4-D culture initiation; the *star* marks the period corresponding to embryogenic character expression and optimal transgenesis ability. *E* embryogenic calli, *NE* non-embryogenic calli

We had previously developed a simple, straightforward regeneration protocol based on mature zygotic embryos as starting material, rather than immature material [10]. This alternative protocol relies on 2,4-D-induced regenerable calli from mature embryos reduced in tissue fragments (Fig. 1). Histological studies were conducted in order to determine the morphogenetic pathway and its tissular origin. The identification of the major events along the SE pathway leading to the genesis of plantlets, the localization of the origin of the process and the identification of the most favorable tissue environment are discussed elsewhere (manuscript submitted). We used this simple and well-characterized wheat regeneration procedure as an experimental model for studying the tissue culture and transformation properties.

Transcription profiling of target genes and enzymatic assays were conducted to gain better understanding of key events underlying the early processes involved in plant neoformation when wheat mature zygotic embryo fragments are 2.4-D induced in culture. We also sought to gain a better understanding of transformation receptiveness: by tracking the physiological evolution of somatic cells during the acquisition of embryogenic competence, we investigated jointly the reasons for the fluctuations in the transformation ability of those cells.

Competence in callogenesis (undifferentiated cellular proliferation, Fig. 1, ‘non-embryogenic callus’) and in embryogenesis (white to green-white, compact and smooth, plane or dome-shaped or nodular appearance calli formed with densely proliferating cells, Fig. 1, ‘embryogenic callus’) was identified. Those calli termed as ‘embryogenic’ tended to engage in differentiation, confirmed by cell cluster greening thereafter.

RNA was extracted from tissues displaying distinct behavior (i.e., embryogenic vs. non-embryogenic) and after varying culture periods (i.e., 2, 6, 8, 14 and 20 days), the latter producing different responses to genetic transformation, on the premise that the 6-day culture is a pivotal period (when the embryogenic character becomes apparent macroscopically, and this coincides with the most suitable physiological state for the particle bombardment-mediated DNA introduction and long-term transgene expression) [9] (Fig. 1).

**Fig. 2:**
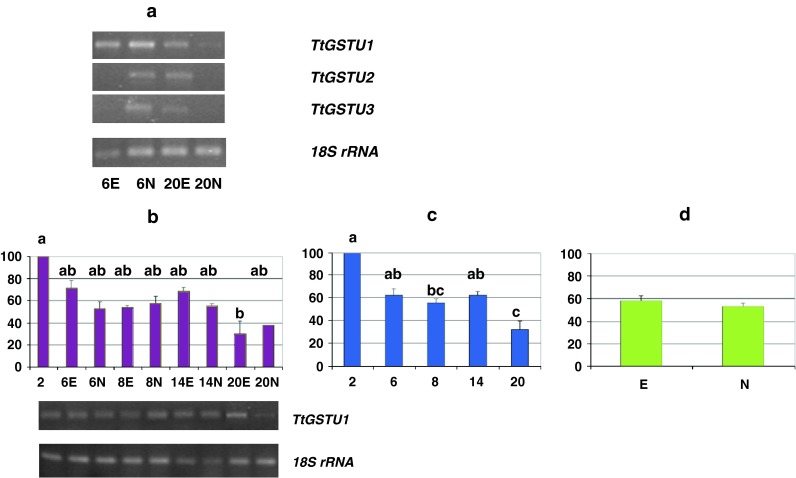
GST transcription profiles—relative abundance of transcripts and representative gels showing their RT-PCR amplification rate. Restricted temporal analysis of the relative transcription of individual GST genes (*TtGSTU1*, *TtGSTU2* and *TtGSTU3*) (**a**), and complete temporal analysis of the *TtGSTU1* (**b**, **c**, **d**); fluctuations in the relative level of transcripts over time and depending on the different tissue types (**b**), average values across the culture duration (**c**) and the morphogenic character displayed by tissues (**d**). *Bars* represent mean values ± SDM (standard deviation of the mean) from at least three independent experiments. Data were analyzed by ANOVA (SYSTAT 8.0) following arcsinrac(x) variable transformation. Means were compared using the Tukey test. Data with different *letters* are significantly different at *p* ≤ 0.05. *2*, *6*, *8*, *14*, and *20* number of days after 2,4-D culture initiation, *E* embryogenic tissues, *N* non-embryogenic tissues

**Fig. 3:**
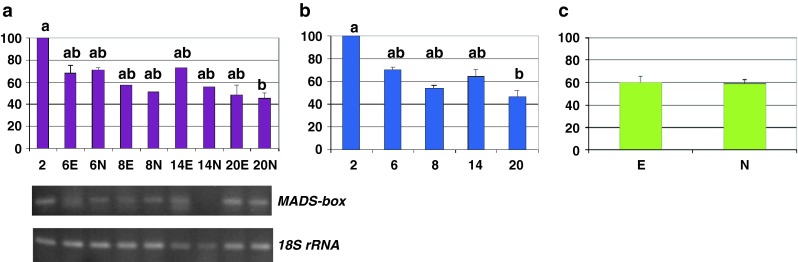
MADS-domain transcription factor, MADS-box transcription profile—relative abundance of transcripts and representative gels showing their RT-PCR amplification rate. Fluctuations in the relative level of transcripts over time and depending on the tissue types (**a**), average values across the culture duration (**b**) and the morphogenic character displayed by tissues (**c**). *Bars* represent mean values ± SDM from at least three independent experiments. Data were analyzed by ANOVA (SYSTAT 8.0) following arcsinrac(x) variable transformation. Means were compared using the Tukey test. Data with different *letters* are significantly different at *p* ≤ 0.05. *2*, *6*, *8*, *14*, and *20* number of days after 2,4-D culture initiation, *E* embryogenic tissues, *N* non-embryogenic tissues

**Fig. 4:**
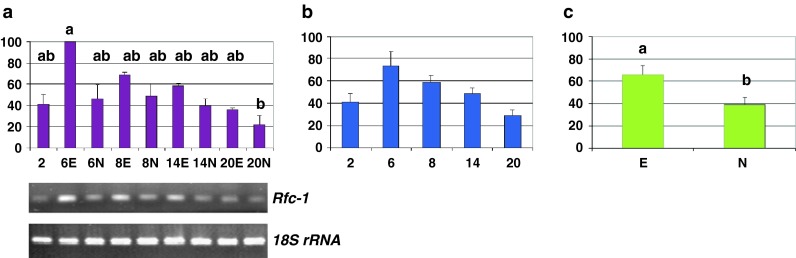
Replication factor C large subunit, *Rfc-1* transcription profile—relative abundance of transcripts and representative gels showing their RT-PCR amplification rate. Fluctuations in the relative level of transcripts over time and depending on the tissue types (**a**), average values across the culture duration (**b**) and the morphogenic character displayed by tissues (**c**). *Bars* represent mean values ± SDM from at least three independent experiments. Data were analyzed by ANOVA (SYSTAT 8.0) following arcsinrac(x) variable transformation. Means were compared using the Tukey test. Data with different *letters* are significantly different at *p* ≤ 0.05. *2*, *6*, *8*, *14*, and *20* number of days after 2,4-D culture initiation, *E* embryogenic tissues, *N* non-embryogenic tissues

**Fig. 5:**
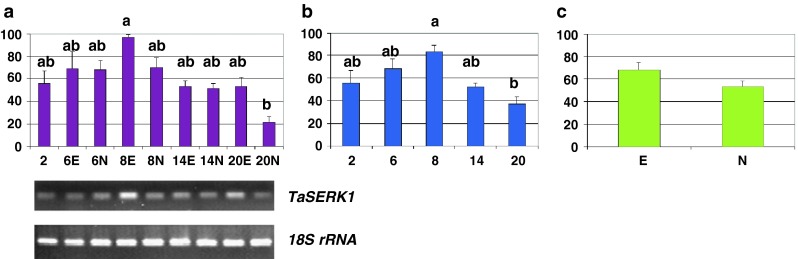
Somatic embryogenesis receptor kinase, *TaSERK1* transcription profile—relative abundance of transcripts and representative gels showing their RT-PCR amplification rate. Fluctuations in the relative level of transcripts over time and depending on the tissue types (**a**), average values across the culture duration (**b**) and the morphogenic character displayed by tissues (**c**). *Bars* represent mean values ± SDM from at least three independent experiments. Data were analyzed by ANOVA (SYSTAT 8.0) following arcsinrac(x) variable transformation. Means were compared using the Tukey test. Data with different *letters* are significantly different at *p* ≤ 0.05. *2*, *6*, *8*, *14*, and *20* number of days after 2,4-D culture initiation, *E* embryogenic tissues, *N* non-embryogenic tissues

**Fig. 6:**
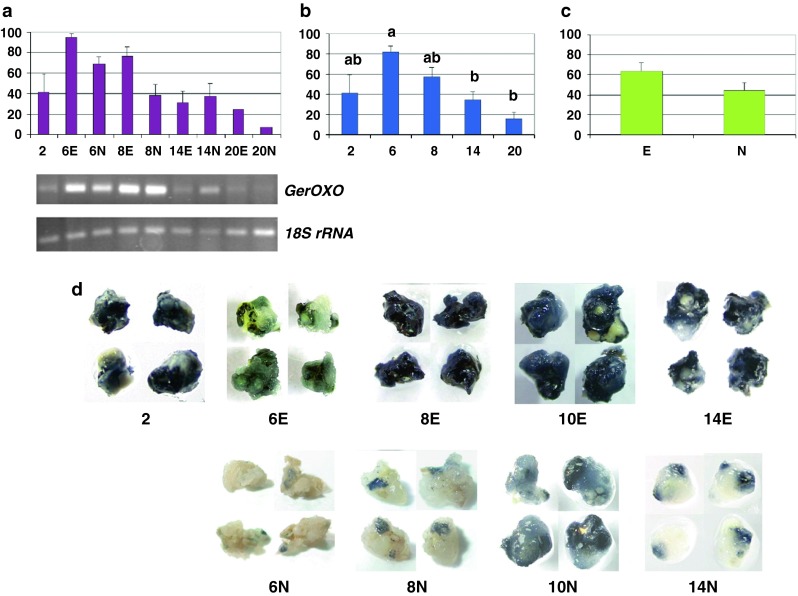
Germin oxalate oxidase, *GerOXO* transcription and enzymatic profiles—relative abundance of transcripts and representative gels, histochemical detection of OXO activity. Fluctuations in the relative level of transcripts over time and depending on the tissue types (**a**), average values across the culture duration (**b**) and the morphogenic character displayed by tissues (**c**). Fluctuation in the OXO activity over time and depending on the tissue types (**d**). *Bars* represent mean values ± SDM from at least three independent experiments. Data were analyzed by ANOVA (SYSTAT 8.0) following arcsinrac(x) variable transformation. Means were compared using the Tukey test. Data with *different* letters are significantly different at *p* ≤ 0.05. *2*, *6*, *8*, *14*, and *20* number of days after 2,4-D culture initiation, *E* embryogenic tissues, *N* non-embryogenic tissues

Semi-quantitative RT-PCR (Fig. 2, 3, 4, 5, 6) was used to determine the relative levels of expression of genes involved in adaptive response to stress (GST), the transcriptional regulation of cellular processes and organ development program (MADS-box), cell proliferation (RFC large subunit), the transition of the somatic cells into an embryonic pattern of differentiation (SERK), the acquisition/maintenance of embryogenic competence and defense (GerOXO). The specific primers are listed in Table 1.

### Glutathione *S*-transferases (GSTs)

The differential transcription of three members of the GST tau wheat family [25] was considered in this study: the two tandemly repeated GST genes, *TtGSTU2* and *TtGSTU1* [GenBank: AY013753] and the related GST-like sequence, *TtGSTU3* [GenBank: AY013754].

As confirmed by RT-PCR analysis, each specific primer pair allowed each *T. aestivum* individual GST sequence to be amplified, and the amplified fragments were of the expected sizes. A higher inducible expression of the *TtGSTU1* gene was observed (Fig. 2a).

An early transcriptional response of the cells submitted to 2,4-D in vitro induction was illustrated by the *TtGSTU1* gene (Fig. 2). In the detailed analysis (temporal and tissue specific measures of mRNA abundance), the level was significantly higher early in the culture (i.e., day 2, *p* = 0.03) (Fig. 2b). In the temporal analysis (values averaged, whatever the morphogenetic character of the tissues), this level was more than 1.9 times greater at 2 days than during the rest of the investigated period (*p* = 0.03). The term of the temporal analysis was marked by a clear decrease (Fig. 2c). In the embryogenic versus non-embryogenic analysis (on average, covering the whole investigated period), the mRNA level of this member of the wheat *GST* family did not differ significantly (*p* ≤ 0.05) (Fig. 2d).

### MADS-box transcription factor (MADS-box)

PCR with degenerate primers was used for cloning a wheat MADS-box cDNA (*T. aestivum* L.) putatively homologous to the maize *ZmMADS1* transcription factor, using RNA extracted from embryogenic cultures as the template (paper in preparation). The partial wheat cDNA (113 bp, see Additional file 1 in Electronic Supplementary Material) showed 100 % homology to the *T. aestivum* *TaMADS#12* mRNA for MADS box transcription factor [GenBank: AB007505] [26] and encompassed the highly conserved sequence motif MADS (DNA binding domain), typically found in this transcription factor family.

This MADS-box gene produced a transcription profile similar to that of the *TtGSTU1* gene (Figs. 2, 3). In the detailed analysis, the mRNA level was significantly higher 2 days after 2,4-D culture initiation (*p* ≤ 0.05) (Fig. 3a). In the temporal analysis, this level was 1.7 times higher, on average, in the 2 day-old culture than during the rest of the investigated period (*p* = 0.03). A progressive decrease was observed temporally (Fig. 3b). The embryogenic versus non-embryogenic analysis revealed no difference between the studied tissue types (Fig. 3c).

### Replication factor C (*Rfc-1*)

In rice the five subunits that make up the RFC (OsRFC) were expressed strongly in proliferating tissues [27].

A search in the public database of the National Centre for Biotechnology Information (NCBI, http://www.ncbi.nim.nih.gov), with the five subunits of the OsRFC ([GenBank: AB109200, GenBank: AB045677, GenBank: AB038319, GenBank: AB066661, GenBank: AB109201] corresponding to *OsRFC1, 2, 3, 4, 5*, respectively) as the query entries, produced only one homologous sequence in wheat, encoding the large subunit replication factor C (*Rfc*-*1* gene, [GenBank: AJ318783]), which shares 92 and 89 % sequence identities (at the nucleic acid and amino acid level, respectively) with *OsRFc1*. This wheat *Rfc*-*1* gene has been shown to be governed by cell-cycle regulation, with mRNA accumulating in cell populations enriched for early S-phase [28].

In the detailed analysis, using primers specific for *Rfc*-*1* sequence, a significantly higher mRNA level than for all other samples was observed on day 6 in the samples identified as embryogenic (*p* ≤ 0.05) (Fig. 4a). In the temporal analysis, a peak was observed in samples collected on day 6, followed by a progressive reduction (Fig. 4b). Lastly, in the embryogenic versus non-embryogenic analysis, a significantly higher level marked the tissues that were embryogenic (*p* = 0.03) (Fig. 4c).

### Somatic embryogenesis receptor kinase (SERK)

At the time that this study was initiated, no gene had been found in searches for sequences annotated as ‘SERK’ or ‘SERK homologous’ in wheat (*T. aestivum*) using the database of the NCBI. The functional characterization of homologous SERKs in Poaceae had been reported in maize [29].

A search in the public database using the coding sequences of *Z. mays* *SERK1, SERK2 and SERK3* as query entries [GenBank: AJ277702, GenBank: AJ277703, GenBank: AJ400870] retrieved two wheat clones, full mRNA sequences [GenBank: BT009426, GenBank: BT009223], with the former sharing 83 % sequence identity with *ZmSERK1* and *ZmSERK2*, 74 % with *DcSERK* [GenBank: U93048] and 73 % with *AtSERK1* [GenBank: A67827], at the nucleotide level.

This clone [GenBank: BT009426] has since been assigned as *T. aestivum* *TaSERK1*. Its expression has been showed to be auxin inducible and to correlate with the initiation of SE in wheat leaf base cultures [30].

In the detailed analysis, a noticeably higher abundance of *TaSERK1* mRNA was observed on day 8 in tissues identified as embryogenic (*p* = 0.02) (Fig. 5a). In the temporal analysis, on average this level peaked significantly in samples collected 8 days after the 2,4-D in vitro induction (*p* = 0.02) (Fig. 5b). In the embryogenic versus non-embryogenic analysis, the tissues involved in an embryogenic differentiation process showed a greater tendency to express the *TaSERK1* gene (20 % more), although this difference did not appear statistically significant at *p* ≤ 0.05 (Fig. 5c).

### Germin oxalate oxidase (*GerOXO*)

In the detailed analysis, the level of *GerOXO* messengers appeared to fluctuate, reaching a maximum value for embryogenic tissues after 6 days (Fig. 6a). In the temporal analysis, their abundance increased over time, marking a significant peak day 6 (*p* ≤ 0.05), after which the mRNA content gradually decreased (Fig. 6b). In the embryogenic versus non-embryogenic analysis, despite a high rate of expression of *GerOXO* in embryogenic tissues, the difference (30 % more) did not appear to be significant at *p* ≤ 0.05 (Fig. 6c).

### Oxalate oxidase (OXO) enzymatic activity

The fundamental nature of the germin family, which is involved in both adaptability and developmental processes, including through SE [21, 31, 32], motivated us to conduct a deeper analysis. Within the wheat germin family, the member considered in this study has received considerable attention and was the object of numerous characterization studies, in both homologous and heterologous expression systems. A clear correlation has been demonstrated between the level of transcription, the synthesis of the protein and the level of its enzymatic activity when this gene was expressed either in wheat [19, 23, 33] or in other plant species (e.g., [34–38]). Taking advantage of the ease of identifying the presence of the corresponding proteins via analysis of their OXO enzymatic function (i.e., readily detectable through an oxalate-dependent histochemical detection of its reaction product (H_2_O_2_) fuelling a peroxidase-linked colorimetric reaction [24, 39]), OXO activity was temporally monitored in 2,4-D induced calli from mature embryo fragments, in tissues growing after 2, 6, 8, 10 and 14 days of culture, either embryogenic or non-embryogenic (Fig. 6d).

Temporally, significant activity observed in the 2-day old calli (the dark color detected) was followed by a transient decrease on day 6 and a peak on day 8 in the tissues engaged in the embryogenic differentiation process. Subsequently, there was a gradual decrease (Fig. 6d, upper series of pictures). These temporal changes tracked through the enzymatic assays accorded with the patterns in the gene transcript levels, but with the kinetics of OXO activity following that of the accumulation of messengers (Fig. 6b). The important enzymatic activity visible in the most responsive 2-day old calli suggests a substantial induction of transcript synthesis during the previous period.

When comparing the embryogenic versus non-embryogenic tissues (Fig. 6d, the upper vs. the bottom series of pictures), specific changes tracked through enzymatic assays confirmed the differential mRNA levels previously recorded (i.e., transcripts were most abundant in embryogenic tissues, Fig. 6c). Embryogenic tissues showed a biphasic colorimetric reaction over time (Fig. 6d, upper series of pictures), but this was always clearly paler in non-embryogenic tissues (Fig. 6d, bottom series of pictures).

**Fig. 7:**
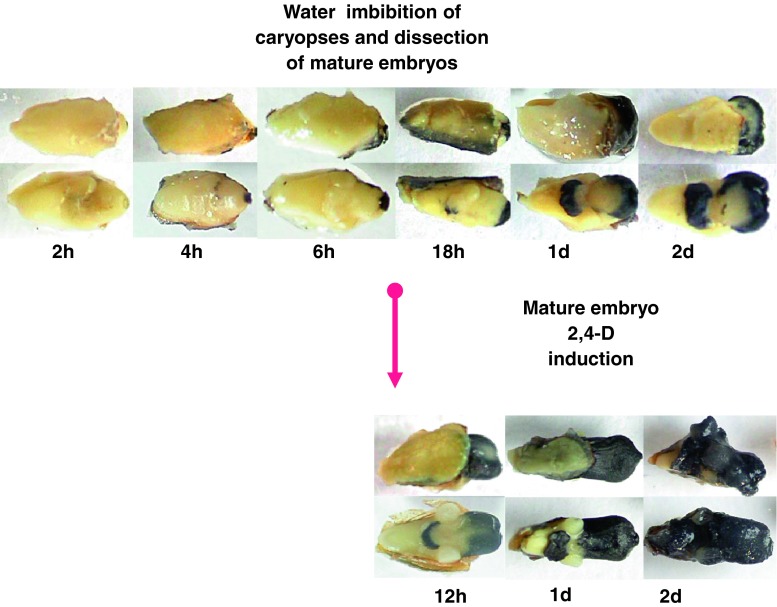
Wheat caryopsis imbibition and entire embryo culture—histochemical detection of OXO activity. Enzymatic activity after increasing the duration of seed imbibition (**a**), and when mature embryos were 2,4-D induced after a 16 h-rehydration (**b**). *Upper alignment* of pictures, embryo scutellum side up; *lower alignment*, scutellum side down. *h* hour, *d* day

In order to complete our analysis of OXO activity, an enzymatic assay was conducted during the mature zygotic embryo culture by applying the same protocol to whole embryos rather than fragmented tissues (Fig. 7). Imbibing seed water alone triggered OXO activity in some embryo tissues, but a synergic effect was observed when these embryos were 2,4-D induced after a 16-h rehydration (i.e., as described in our in vitro culture procedure [9]). After a 2-day 2,4-D treatment, the visible dark color that indicated important enzymatic activity had extended to the remaining tissues.

## Discussion

To gain a better understanding of the molecular events involved in the acquisition of competence for gene transfer and tissue culture, target gene expression patterns were profiled.

The GST, MADS-box, RFC, SERK and GerOXO gene families were considered promising for learning more about the chronology of the critical cellular processes, such as the adaptation to the in vitro context, regulation of the cellular fate, induction of cell division, and their commitment in the embryogenic pattern of development.

Tissue samples were randomly collected at each of five time points after the 2,4-D induction (day 0): (i) one time point during cell proliferation initiation and callus induction (day 2); (ii) two time points during callus development and around the acquisition of the embryogenic character and transformation ability (day 6 and day 8); and (iii) two time points during further differentiation and morphogenetic development (day 14 and day 20). Where possible, the tissues visually engaged in the process of embryogenesis were analyzed separately from those that were not (after day 6, a crucial period in terms of both the macroscopic manifestation of the embryogenic character and the physiological receptivity to the introduction of transgenes) (Fig. 1).

### In vitro culture as stress signal (GSTs)

A profusion of physiological and metabolic adjustments occur in response to a multitude of stress factors of a physical and chemical nature when plant tissues are subjected to in vitro culture conditions [13].

“Somatic embryogenesis is the process by which somatic cells, under induction conditions, generate embryogenic cells, which go through a series of morphological and biochemical changes that result in the formation of a somatic embryo” [40]. Starting with de-differentiation, SE is a multi-step, complex and highly regulated process that occurs as part of natural plant development in vivo (e.g., apomixis, a form of asexual reproduction, [41]) or is achievable in plant tissue culture [14]. The ability of several somatic cell types to engage in embryogenic development illustrates their adaptive response to the in vitro environmental constraints. This response includes the re-programming of gene expression in parallel with stress signaling cascade activation and defense response induction [13].

Gene expression analyses at both the proteome and transcriptome levels have led to the identification and characterization of many stress-related genes and proteins associated with the early stage of SE [42, 43]. Belonging to the antioxidant enzyme system, GSTs (EC 2.5.1.18) form a superfamily of multifunctional proteins, playing an important role in the overall natural defense mechanisms in all living organisms, whose expression is induced after exposure to a multitude of stresses (for review, see [44]).

Plant GSTs respond to a wide range of agents and conditions in which increased level of ROS production is a common factor (reviewed by [45]). Biotic and abiotic stresses (pathogens, natural and synthetic toxins, heavy metals, heat shock), ROS themselves and phytohormones activate the transcription of individual GST genes differentially [46].

Beyond their functions associated with detoxification and other stress responses in plants, recent studies show that several GSTs also play a role in normal plant growth and development in vivo, and in regeneration in vitro [22, 47, 48]. Lastly, in the interacting molecular network triggered very early (the first 24 h) during the auxin-induction phase in wheat leaf base segments, the GST protein family emerged as one of the three major nodes [16].

The GSTs of the Phi and Tau classes are plant-specific and are the most abundant subclasses in plant tissues. Besides their well-known conjugating activity to exogenous compounds both classes possess GSH-dependent peroxidase activity (GPOX) which uses GSH to reduce organic hydroperoxides. Also observed for several wheat tau GSTs [49] the latter activity is of particular significance for the prevention of hydroperoxide-induced damages (reviewed by [45, 50, 51]). The primers we used were designed to selectively amplify three members of the GST Tau wheat family (i.e., the two highly homologous genes, *TtGSTU1* and *TtGSTU2,* and the GST-like sequence named *TtGSTU3*) for expression studies in *Triticum* species in terms of their biochemical function in herbicide metabolism [25].

In Xu et al. [25] gene-specific semi-quantitative RT-PCR analyses, the tandemly duplicated *TtGSTU1* and *TtGSTU2* genes (but not the *TtGSTU3* sequence) were induced in root and shoot tissues by the synthetic auxin 2,4-D. *TtGSTU1* expression was always higher than that of *TtGSTU2.* Both TtGSTU1 and TtGSTU2 proteins were thought to have important roles in xenobiotic metabolism in wheat, and might also have significant yet undefined roles in response to plant stresses [25].

In a pilot assay, we initially assessed the selective amplification of each individual transcript for two-sample tests (i.e., two time points, days 6 and 20) (Fig. 2a). In our results we recorded the amplification of the TtGSTU3 sequence in embryo tissue cultures. The expression of this gene had not previously been detected by Xu et al. [25] whose analyses were performed on shoot and root tissues. The *TtGSTU1* transcripts were more abundant, as observed earlier [25], and we followed the transcription profile of the latter more closely (Fig. 2b–d).

Unique combinations of often multiple interactive signaling pathways (i.e. various phytohormones, ROS or antioxidants) produce the distinct transcriptional activation patterns of individual GSTs during stress (reviewed by [46]). An early response of wheat cells submitted to 2,4-D in vitro induction was illustrated by a quick transcriptional activation of the *TtGSTU1* (Fig. 2b, c), reflecting the role of this gene superfamily in the immediate response of plants to environmental alterations and constraints.

*TtGSTU1* expression could be triggered either through an overall response to stressful in vitro culture conditions, associated with a global deterioration of the environment (i.e., via the central role of GSTs in antioxidant functions and defense mechanisms), or through a 2,4-D specific induction as a growth regulatory substance (putative ABA-, ethylene- and, in particular, auxin-responsive regulatory elements were identified in the promoters of those GST genes [25]).

**Fig. 8:**
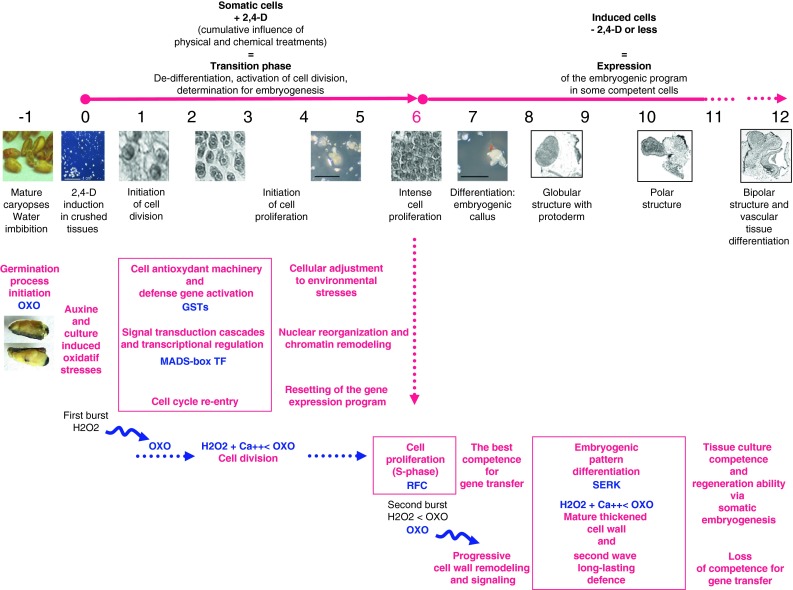
Events surrounding the acquisition of competence in tissue culture and genetic transformation: an integrated approach of data and concepts at the tissular, cellular and molecular levels. With wheat as the observed biological system, this model summarizes data and concepts aimed at providing an analytic-to-holistic view of the regenerative property of plant cells and their receptivity to direct gene transfer. Cells are complex and dynamic systems that go through a sequence of physiological stages along the regenerative pathway. The molecular signatures of the responses to stress/auxin and the release of embryogenic development are integrated with the series of microscopic/macroscopic observations, together with the cellular processes that might interfere with transformation ability over time (time scale in days, −1 to 12, relative to embryo culture initiation). In particular, the germin oxalate oxidase gene family provides an appropriate spatio-temporal molecular signature of the successive stages of both competencies in cereals. (1) Both the higher level of intracellular H_2_O_2_ and the activity of cellular antioxidant machinery have been shown to be crucial for cell division and the expression of totipotency [161–163, 196, 197]. (2) An initial oxidative signaling (a rapid and transient production of huge amounts of ROS) induces antioxidant defenses. Notably, belonging to the antioxidant apparatus, plant GSTs, with GSH, play a central protective role in counteracting oxidative stress [198], sensing and maintaining redox homeostasis [199]. A growing number of studies recognize the importance of the redox environment for growth and development, and of the GSTs link with the early developmental phases of SE (reviewed in [200]), making GST transcript abundance an early signal for the identification of embryogenic cultures. (3) An acclimation response is complex and occurs in different phases. Immediately after the cellular changes that occur as a result of stress exposure, defense processes are triggered; the concomitant activation of a set of specific genes is indicative of the adaptive behavior of cells adapt [201]. Reprogramming the gene expression pattern is achieved by regulation at the transcriptional level, as could be reflected by the activation of the MADS-box TF, probably involved in the integration of environmental and developmental cues. (4) Re-entry into the cell cycle plays a crucial role in the expression of cellular totipotency and in the transformation competent state [11, 92]. Strongly expressed in proliferating tissues, an increased Rfc transcript synthesis is on average correlated with the highest response to embryogenesis, as expected. But this critical component of the DNA replication machinery is cell-cycle regulated and was also used as a marker of S phase-enriched cell populations in our study [28]: the Rfc transcript level marks a sharp increase in the 6-day-old proliferating cells that are the most responsive to receiving and stably expressing transgenes [9]. Histological observations show that the physiological state reached at day 6 corresponds to a pivotal developmental window between undifferentiated cell proliferation and organization, constituting a transition phase between two different dynamic regimes. (5) First discovered as a marker of the transition of the somatic cells into an embryonic pattern of differentiation, SERKs were shown to confer embryogenic competence. As previously observed [104], SERK maximal transcription occurred at the globular stage of SE differentiation. The SERK homologue considered in this study is auxin inducible and calcium dependent. It is expressed rapidly and long before the first appearance of somatic embryos [30]. (6) All along the steps of the regenerative process, among the stress responses, the accumulation of germin OXOs appears to be of particular interest. Involved in SE, these defense-related proteins are H_2_O_2_ inducible, and they themselves produce Ca^2+^ and H_2_O_2_ from oxalate under acidic conditions. The release of Ca^2+^ and H_2_O_2_ has long been considered as the biochemically important result of their OXO activity. Both these molecules play key roles in cell elongation and division, in relaying a stress signal, in cell wall reinforcement and in establishing a second-wave long-lasting defense (see “Discussion” section). After the transient first burst that occurs immediately after an abiotic stimulus, a second oxidative burst of H_2_O_2_ resulting from OXO activity would be involved in long-lasting adaptive behavior and constitutive defense, as observed earlier in an another member of the Poaceae family [149, 152]. H_2_O_2_ and Ca^2+^ are known to be interconnected signaling molecules that are essential in the multiple pathways of plant innate immunity [192]. During the *Agrobacterium* transformation process, successful DNA transfer is possible due to the ability of *Agrobacterium* to hijack fundamental cellular processes and to interfere with plant immunity pathways in order to counteract host defense [181, 182]. According to the temporal–spatial concept of ROS wave signaling [178], OXOs appear as attractive candidates in propagating the ROS wave throughout the cells, carrying the signal in time and in feeding the cascade of cell-to-cell communication events and over time

*TtGSTU1* messengers were the most abundant when cell proliferation was initiated (2 days, Fig. 8). Cell proliferation and active aerobic metabolism occurring upon auxin SE induction is associated with a burst of reactive oxygen species, including hydrogen peroxide [52, 53].

This result could also be interpreted in terms of the role of the synthetic auxin 2,4-D itself, which is still a matter of investigation: it is a key substance for efficiently inducing embryogenic competence (i.e., as a growth regulator that mimics auxins, with a concentration-dependent mode of action, triggering a specific developmental adaptive response), but is also known for its herbicide and therefore phytotoxic activity, and possibly acts as an individual stress factor (i.e., the physical or chemical stress factors by themselves are known to be SE inductors) (reviewed by [13, 54]).

Lastly, in contrast to Singla et al. [16], the transcripts were identically induced in tissues, whether embryogenic or not (Fig. 2b, d); this suggests that TtGSTU1 was a protein acting at the very early phase during the auxin induction in this study, with wheat mature embryo fragments as a culture system.

### Transcriptional regulatory network: release of the embryogenic program (MADS-box)

During the somatic-to-embryogenic transition period, chromatin remodeling is implicated in the coordinated reorganization of the cellular state [13, 55]. Dynamic changes in chromatin structure are central for the reversion of the differentiation process, leading to a resetting of the gene expression program and activation of silent genes, and the release of the embryogenic program otherwise repressed in vegetative plant cells [55–57].

Signal transduction cascades are still largely unexplored, but the presumed increased activity of the various signaling pathways is assumed to be associated with a differential expression of many families of transcription factors (TFs).

TFs are master regulators of developmental patterns, in both animals and plants [58–60], notably in regulating different aspects of embryonic processes in higher plants [61–64].

The MADS group members include many examples where the change in activity of a single TF has been shown to have a profound effect on an important aspect of plant biology [65–71].

Among them, MIKC-type proteins are plant specific and the most characterized MADS group in plants [69, 72]. In dicot species, the *AGAMOUS*-*like 15* (*AGL15*) gene is highly expressed only in cells that take on an embryonic identity, whatever the embryo origin (i.e., zygotic, somatic, apomictic) [63, 73]; and its constitutive expression promotes somatic embryo development [74, 75].

There is little information about the implication of the members of this very large MADS-box gene family in the in vitro embryogenic process in Poaceae. Nevertheless, within the molecular network of the genes shortly expressed (24 h) in 2,4-D-induced wheat leaf bases, genes involved in the transcription machinery formed one of the largest groups, and included the MADS-box protein family [16]. In maize, a member of MADS-box family of type II (MIKC) has been functionally characterized. *ZmMADS1* [GenBank: AF112148] was shown to be specifically induced in cells able to form somatic embryos (i.e., during early SE between the embryogenic competent cell stage and the globular phase), but not to be expressed in non-embryogenic suspension cultures neither in immature or mature zygotic embryos [76].

Based on sequence information from *ZmMADS1* and given the high degree of synteny among species sharing a common evolutionary history in the cereal lineage, we designed degenerate primers surrounding the conserved MADS-box region and isolated a partial cDNA from wheat, representing a putative *ZmMADS1* orthologous.

The MADS-box genes constitute a large multigene family dispersed throughout the genome in wheat [26, 77–79]. This is the first report of a spatio-temporal analysis of a MADS-box gene during SE in wheat. A differential expression level of this sequence was detected temporally (Fig. 3b), but not according to the type of tissue analyzed (Fig. 3c): the transcripts appeared at the early stage of the culture induction, and then gradually decreased (Fig. 3a, b).

It is worth mentioning that MADS-domain TFs characterized in wheat have been shown not only to be involved in plant developmental control [77–79], but also to be differentially expressed in stress-induced tissues [80–82] and to act at the confluence of the integration of environmental and developmental signals [83]. Stimulation of transcript accumulation at the time of the culture initiation (Fig. 3b) suggests that the cloned cDNA could be involved in early embryogenesis. It could also be involved in stress response associated with the conditions of in vitro survival, or both. An overlap between functions of genes involved in development and stress response has been documented in rice [70, 84] and wheat [85].

One could also explore a possible evolutionary functional variation between the cloned sequence and that of maize used as a reference. This important and complex MADS family has greatly expanded during the evolution of land plants [86, 87]. In particular, the MIKC-type proteins display an unusually efficient and high degree of evolutionary plasticity and functional diversification [88]. This could be especially true for wheat, as the evolutionary history of its genome is considerably richer in multiple events of large-scale duplication and polyploidization, stigmata of the evolution of Poaceae and the allohexaploid bread wheat, in particular.

Finally, high TF transcriptional activity at the beginning of tissue culture could simply mark the competent chromatin state associated with the reprogramming of the gene expression pattern [89–91].

### Somatic embryogenesis and transgenesis: re-entry in cell cycle as a crucial step (RFC)

The production of nuclear transgenic plants requires the delivery of foreign DNA into the nucleus and its stable insertion into the host genome, followed by the multiplication and differentiation of transformed cells into organs or embryos, culminating in the subsequent regeneration of whole plants [92]. Transgenic plants can be obtained only from those cells that are competent for both transformation and regeneration, with efficiency depending on the degree of overlap between both types of cells [12].

The regeneration and transformation ability of cells are closely linked to their proliferation potential.

Regenerative ability depends largely on the ability of somatic cells to undergo dedifferentiation, a process preceding re-entry into the cell cycle, trans- or redifferentiation [56, 57]. In plants, the remarkable cellular plasticity is exemplified by the reversion of certain mature cells to a stem cell state and the regeneration of the whole plant [93]. This extraordinary feature requires cell-cycle reactivation at the G1-S transition phase, with the G1 phase that is central to the integration of signals that regulate the reactivation of cell proliferation or the exit from the cell division cycle to differentiation [94], and with the S-phase (DNA synthesis) culminating in full genome replication.

Actively dividing cells are the most responsive targets for transgene nuclear insertion [95, 96], including via particle bombardment [96–98]. A strong positive correlation was also observed between the cell cycle phase at the time of DNA transfer and A*grobacterium* transformation efficiency. This pivotal importance of S-phase control functions for the *Agrobacterium*-mediated stable integration mechanism has been independently demonstrated previously [99–102]. To our knowledge, no such clear developmental control (cell cycle control and progression) has been established for direct DNA transfer strategies, notably via biolistic DNA delivery.

The large subunit of the wheat RFC considered in this study (*Rfc*-*1,* [GenBank: AJ318783]) is one of the essential components of the cell DNA replication machinery, its expression has been shown to be subjected to cell-cycle regulation, with a peak in early S-phase [28].

*Rfc*-*1* messengers were most abundant in the 6 day-old calli that were embryogenic (Fig. 4). As in the case of the rice RFC homologue [27], *Rfc*-*1* messengers were more abundant in the proliferating meristematic cells (day 6, Fig. 8). This is consistent with the fact that actively dividing cells are essential for establishing a new developmental program, organ morphogenesis and, in this case, somatic embryo completion [13]. *Rfc*-*1* messengers were also more abundant (Fig. 4b) in tissues shown to have the best stable transformation ability (6-day-old tissues, [9]), which accords with the fact that dividing cells are the most effective targets for transgene insertion [95–97]. These findings therefore support the view mentioned earlier that the re-entry into the cell cycle has a crucial role in the expression of cellular totipotency [94] and also in gene transfer, nuclear integration and stable expression [92, 99–102].

However, the *Rfc*-*1* transcripts were less abundant after 6 days (Fig. 4a, b). With the embryogenic developmental program being a continuous process, a proliferation state is still needed to some extent for its achievement at this time (see successive stages of development since day 8, Fig. 8). Given that the wheat *Rfc*-*1* gene is cell-cycle regulated, this result suggests that a higher proportion of cells would be S phase-synchronized on day 6 of culture (i.e., during the pivotal period that coincides with the most suitable physiological state for DNA bombardment and long-term transgene expression) [9]. A previous study of the influence of the cell cycle on the efficiency of direct DNA transfer in the synchronized cultured cells of tobacco (Bright Yellow-2) reported that cells bombarded at the M and G2 phases gave higher transformation efficiency than those bombarded at the S and G1 phases, but in the absence of evidence in discriminating between transient and stable expression of the marker gene [103]. Therefore, as demonstrated for A*grobacterium*-mediated transformation [99–102], in our study there appeared to be a positive correlation between the cell cycle S-phase at the time of biolistic DNA transfer and the stable transformation efficiency.

### SERK: marker for SE differentiation and transformability

Among the genes whose expression pattern is altered during early SE induction, the SERK is considered to play an essential role in the vegetative-to-embryogenic transition. First characterized in *Daucus carrota* (carrot) from auxin-induced embryogenic cell cultures, *DcSERK* encoded a leucine-rich repeat (LRR) transmembrane receptor-like kinase (RLK) and was found to mark unequivocally the transition to embryogenesis [104]. Its expression was specifically and transiently expressed during the initiation of embryogenic development up to the globular stage, in suspension cell cultures, initially in the single cells that were able to form somatic embryos, and then during the initial phases of their development. In addition, during zygotic embryogenesis, the expression was detectable transiently in young zygotic embryos of up to 100 cells [104, 105].

SERK homologues were identified in others species, both dicotyledonous and monocotyledonous plants. As in carrot*, homologous* genes were shown to be expressed in the embryogenic cell cultures and somatic embryos [29, 106–110].

Arabidopsis *AtSERK1* ectopic expression has been reported to confer embryogenic competence in culture [106]. Its expression was characteristic of those cells capable of rapid response to hormonal signals and able to form somatic embryos or embryogenic cell cultures [106, 111, 112].

The corresponding protein would be a component of an embryogenesis-signaling pathway. Competent cells might contain an inactive receptor, which is activated by the presence of the proper ligand to switch on the embryogenic program. SERK1 is a component of plasma membrane LRR-RLKs receptors organized into heterooligomeric protein complexes and involved in the brassinolide signaling pathway [113, 114]. SERK1 appears as an active member in the extracellular signal perception, followed by intracellular signal transduction via phosphorylation of specific targets that results in the altered expression of hundreds of genes [114–117].

Three SERK genes have been isolated from wheat and characterized. The signaling of 2,4-D-mediated induction of SE in leaf tissues was mediated by the SERK pathway, of which *TaSERK1* is one component [16, 30].

In this study, there was a significantly higher relative abundance of *TaSERK1* transcripts in tissues identified as embryogenic on day 8 of culture (Fig. 5a).

On average throughout the investigated period (Fig. 5c), a measurable higher mRNA level was observed in calli annotated as embryogenic, although transcripts were also detectable in the other samples. A broader expression pattern than that of *DcSERK* and *AtSERK* had previously been observed in other species [29, 118]. Actually, SERK homologue expression not only marks cells able to form somatic embryos, and is involved in conferring this embryogenic competence, but they also may have a wider developmental role (i.e., including zygotic embryogenesis, apomixes, in vitro organogenesis) [106, 108, 110, 119–121]. SERK proteins are involved in the general mechanisms of biotic and abiotic stress perception. They might partially mediate defense signal transduction for disease resistance response, in addition to their basic role in SE [107, 122–124].

Based on the spatio-temporal analyses, a higher transcript level was measured in the 8 day-old embryogenic structures (Fig. 5a, b), coinciding with the globular stage of embryo development (see histological picture day 8, Fig. 8). After that, the transcript level gradually decreased (Fig. 5b). This finding accords with results observed for carrot, where *DcSERK* expression is characteristic of embryogenic development up to the globular stage and stopped thereafter [104].

It is worth noting that this globular developmental stage coincides with the appearance of the first signs of differentiation (i.e., histogenesis begins with the demarcation of a protoderm covering the globular embryo, Fig. 8, day 8). On the premise that, along with differentiation, cells exit the mitotic cycle [125], this finding is congruent with the lesser transformation ability of wheat calli for culture periods exceeding 6 days [9]. In addition, after 6 days, clusters of dividing cells are enclosed within a structure bounded by cells that differentiate (the protoderm**)** in terms of their future basic protective function (the epidermis). Knowing that most DNA-coated particles penetrate only the epidermal and first few sub-epidermal layers [126], this configuration is a further constraint to the success of the physical DNA transfer.

### Cell wall GerOXO: central role in development and defense

The cell wall is a highly dynamic entity and a fundamental structural element at the heart of the life strategy of plants, their development and defense [127–131].

The processes of wall assembly and remodeling, along with apoplastic metabolism, would be expected to play an important role during both somatic embryogenesis and direct DNA transfer strategies.

First, during in vitro culture, the auxin-induced structural changes of the plant cell wall associated with cell division cycle have long been known. Those changes prelude to cell enlargement preparing for mitosis and later to cytokinesis, involving the deposition of a new, diffusely fibrillar primary wall [132].

Second, cell wall formation in higher plants is central to cellular differentiation and morphogenesis. Its architecture and remodeling reflect functional differentiation of the cells [127], including through SE [133]. As part of a supracellular structure, called the cytoskeleton–plasma membrane–cell wall continuum [128], the cell wall (ECM: extracellular matrix) plays a crucial regulatory role during embryogenesis induced in vitro (for review, see [133]). Detailed temporal reports of ultrastructural changes also show that during the transition from the meristematic to the embryogenic state, ECM remodeling progressively leads to the physical and physiological individualization of single embryogenic cells from their surroundings (closure of plasmodemata, which breaks the symplasmic continuum, callose deposition and local cell wall thickening) and, later, of the globular unit formed through successive divisions of the initial embryogenic cell [134, 135].

Third, during genetic transformation experiments, plasma membranes and the walls of some cell types are the first biological barriers to be pierced or to pass, depending on the biological system being targeted and the cell compartment one wants to attain (nuclear, mitochondria or chloroplast organelles in eukaryotic cells) [95, 98, 136–142]. Plant cell differentiation leads to the increased mechanical strength of the plant wall [130]. Depending on the mode of particle acceleration, the momentum of particles can be more finely controlled, however, the degree of penetration will depend on the thickness of the cell wall [98], which is an effective penetration barrier [143].

In all these contexts, among the proteins associated with enzymatic cell wall metabolism, the apoplastic oxalate oxidases (OXOs) are particularly remarkable given the diversity of information in the literature supporting their implication in biosynthesis, remodeling and reinforcement of plant cell wall structures [144], but also in developmental reprogramming and differentiation [23, 145–148], including via SE [19, 22, 146], and in stress and defense responses triggered by biotic and abiotic aggressions (for review, see [21]).

OXOs (the so-called germin OXOs or simply germins) found in the ‘*true cereals*’ (barley, maize, oat, rice, rye, wheat) and ryegrass are able to break down oxalate liberated from calcium oxalate crystals dissolution [149–151]. The OXO oxygen-dependent degradation of calcium oxalate leads to the release of H_2_O_2_ and Ca^2+^. They are defense-related and H_2_O_2_ inducible [32, 152, 153]. They have been considered as a main provider of H_2_O_2_ in oxalate-producing plants [146, 152] and would also be an important provider of Ca^2+^ [144, 145, 154]. Both these small and diffusible molecules behave as signals or ‘second messengers’ at lower concentrations [155, 156] and are implicated in the biochemistry of the ECM at higher concentrations [144]. They are known to be key elements for cross-linking plant cell wall components; they are required to synthesize new walls during meristematic growth and for wall-stiffening in differentiated or stressed cells [144, 145, 151, 157, 158].

The OXO gene considered in this work responded to a wide range of biotic and abiotic factors in wheat, and its heterologous expression conferred resistance to bacteria, fungi or herbivorous insects [33–38].

In our study, a particular feature of this OXO enzymatic function was its biphasic nature apparent during the observation period (Fig. 6d), with the kinetics of enzymatic activity following that of the accumulation of messengers (Fig. 6b). A similar delay in the kinetics of accumulation of OXO proteins following that of the accumulation of messengers in response to auxin was observed by Caliskan et al. [19] in wheat immature embryo cultures, the time-lag there being about 20 h.

The first peak of OXO activity observed within 2 days of culture initiation (Fig. 6d) coincided with the first signs of auxin-induced proliferation and growing observed macroscopically [10] and confirmed by histological observations (Fig. 8).

This result accords with the analysis of the promoter structure of the wheat *GerOXO* gene: found to contain several auxin-response elements [159], this promoter was auxin-inducible in transgenic plants [160].

This result also accords with the previously established implication of H_2_O_2_ and Ca^2+^ in ECM during meristematic growth: in totipotent protoplasts [161–163], in the actively dividing and expanding cell [164], and during auxin-induced SE in gymnosperm [165, 166].

With regard to the appearance of this first peak, it is worth recalling that the OXO gene product considered in this study had long been known to be a protein marker of the onset of growth in germinating wheat (‘germin’, [167]) following rehydration. The exposure of immature embryos to auxin is also known to trigger OXO protein accumulation [19, 148]. The embryos treated according to our protocol had been subjected to water imbibition before being exposed to 2,4-D induction. The conjunction of these two treatments led to an intensified OXO activity after 2 days of 2,4-D exposure (Fig. 7). This result coincides with the burst of ROS (H_2_O_2_) associated with cell proliferation and active metabolism, during germination [52, 53, 168] and auxin SE induction [52, 53].

The second peak of OXO activity, evident at 8 days (Fig. 6d exclusively in the responsive embryogenic cultures), was displayed by tissues that were embarking on the embryogenic differentiation process at that time, in accord with the first signs of SE differentiation identified histologically that appear at the globular stage (the demarcation of a protoderm on day 8, Fig. 8).

This result accords with several studies showing specific transcription profiles of germins/germin-like proteins (GLPs) during the study of SE in wheat and gymnosperms [19, 165, 169, 170]. In wheat immature embryo culture, there is a clear correspondence between the bulk of the OXO activity found within the regenerating calli of nodular appearance (i.e., in small, densely cytoplasmic cells forming organ primordial) occurring after 7 days and the subsequent organogenic development [19]. In gymnosperms, specifically expressed at the early globular stage [165], the requirement of GLP expression for the maturation and normal development of somatic embryos was established by gene silencing: in silenced lines, the efficiency of maturation is reduced and most of them were blocked at the pre-globular stage [171].

This result also agrees with the presumed structural changes of the plant cell wall associated with differentiation and with the biological function of H_2_O_2_ and Ca^2+^ in mature walls during growth arrest and rigidification [151, 158].

Our results illustrate once again the apparent paradox associated with germin OXO proteins coupled with the increase in cell wall extensibility in cereals [144, 147, 172], but also with terminal growth arrest in differentiating structures [23, 24, 144, 148] and with wall reinforcement triggered in response to biotic and abiotic aggressions (reviewed by [21, 173]).

Regarding receptivity to gene transfer, the cell structure remodeling, derived from OXO enzymatic activity, could be expected to affect the success of DNA delivery through particle bombardment. The changes of cellular ultrastructure, particularly in terms of thickness, composition (callose and pectin) and hence the rigidity properties of the wall, reported to occur during somatic-to-embryogenic transition, are likely to account for much in cell receptiveness to the physical DNA introduction into the nucleus.

In addition, OXO proteins are also apparently deeply anchored in the defense strategy of monocot plants. Some OXOs and GLPs have now been recognized as members of pathogenesis-related protein families typical of monocots [174, 175]. More importantly, convergent literature data suggest that, together with some members of GLPs, the OXOs seem to form the foundation of a basal defense mechanism in Poaceae, as both immediate and long-lasting responses [33, 149, 152, 153, 176, 177].

According to the temporal–spatial concept of ROS wave signaling [178], OXOs could be attractive candidates in terms of relaying and feeding the cascade of cell-to-cell communication events over time. The study of the response to wounding [152] supports the early hypothesis put forward by Berna and Bernier [33] that OXOs are involved in stress-induced signaling. According to Le Deunff et al. [152], in line with a biphasic model, OXO proteins might represent a second wave and an alternative enzymatic pathway for relaying protection mechanisms and for establishing long-lasting defense and adaptive behavior.

It is worth noting that the establishment of plant defense mechanisms has been recognized as an efficient obstacle against an effective A*grobacterium* transformation. During the transformation process, differential gene expression analysis demonstrated a reverse correlation of the efficiency of transformation with the expression level of the host defense genes [179–182]. Further, the fate of the transgene depends on the plant’s diverse adaptive defense mechanisms that act ordinarily on natural foreign, parasitic or ‘invasive’ nucleic acids (such as transposable elements, viroids, RNA and DNA viruses, and bacterial DNA) with a number of parallels with the immune system of mammals [183–185]. We suggest that the establishment of defense mechanisms might exert a prominent influence on the success of any genetic transformation process.

Lastly, our results lend credence to the idea that germin OXOs are ideally suited to playing a part in both initiation and termination of wall expansion [144], as well in SE development [19], in relaying the culture-induced oxidative burst [22, 152] and in the developmental adaptive response ensuring survival by producing H_2_O_2_ and Ca^2+^ [149, 186]. Taken together, this makes these OXO proteins critical enzymes in cereal embryogenic cultures with a potentially significant role in the receptivity of cells to genetic transformation mediated by biolistic delivery.

### Integrated discussion

In the attempt to produce a synopsis (Fig. 8), we propose to integrate the elements and the data gathered in this study at the tissular, cellular and molecular levels together with the experimental data and theoretical aspects from the literature in order to address the question of tissue culture and transgenesis more broadly.

Stress in plants could be defined as any change in growth conditions that alters or disrupts metabolic homeostasis. This requires an adjustment of metabolic pathways, aimed at achieving a new state of homeostasis, in a process that is usually referred to as acclimation [187–189]. Regardless of the nature of biotic and abiotic stimuli, one of the consequences of stress is an increase in the cellular concentration of ROS that are subsequently converted to H_2_O_2_ [190].

A close connection, or an overlapping, between in vitro embryogenesis and stress response pathways has often been highlighted [13–15, 40]. Induction of SE is usually achieved by a stress and/or hormone treatment of somatic cells [14, 15, 43]. Activation of key regulators of embryogenesis is preceded by the stress-induced reprogramming of cellular metabolism [191]. H_2_O_2_ scavenging inhibits not only the activation of cellular defense reactions, but also cell division [163].

Figure 8 summarizes the successive treatments applied to plant tissues, as well as their effects (i.e., the renewed growth when wheat embryos are germinated in water, followed by the abrupt suppression of the sprouting of the embryonic axis, its wounding and torn tissues exposed to the physico-chemical/hormonal in vitro environmental context), which collectively disrupt metabolic homeostasis and cause stress, triggering a first oxidative burst (Fig. 8, auxin and culture induced oxidative stresses, first H_2_O_2_ burst). ‘Oxidative stress’, formerly a pejorative term denoting a harmful process to be avoided, actually represents a positive process known as ‘oxidative signaling’ [192], an essential component of the repertoire of signals that plant cells use to perceive the environment and make appropriate metabolic/physiological adjustments. ROS participate in signal transduction cascades in processes as diverse as mitosis, growth, development and adaptation. Intimately interconnected with calcium signaling, ROS/redox signaling/antioxidant interaction is seen as a switching node in plant cell physiology [192–195]. Through crosstalk with phytohormones (mainly auxin and ethylene) and sugars, this convergence hub between stress signaling and development allows specific responses to environmental in vitro challenges to be orchestrated [11] (Fig. 8, cellular adjustment to environmental stresses). Dynamic changes in chromatin structure are central to the reversion of the differentiation process and the developmental transition. The developmental switching in somatic cells towards the embryogenic pathway is a release from suppression, controlled at the chromatin level, through a global and dynamic reorganization of the chromatin structure, giving the somatic cells the ability to manifest the embryogenic pathway [14, 15] (Fig. 8, nuclear reorganization based on chromatin remodeling and resetting of gene expression program). During the ongoing SE process, after dedifferentiation, cell division reactivation is the second requirement for pluripotentiality, before the coordinated proliferation process progressively culminates in the development of organized structures (Fig. 8, cell cycle e-entry, followed by cell proliferation and embryogenic pattern differentiation). Meanwhile, a dynamic process of ROS signaling is thought to occur within and between cells, relaying defense- and stress-adaptive processes [178]. It is worth recalling that, during the *Agrobacterium* transformation process, repressing the host defense response is prerequisite to successful transformation. Differential gene expression analysis demonstrated a reverse correlation of transformation efficiency with the expression level of the host defense genes, notably the GSTs [179–182].

Figure 8 provides an integrated view of the functional links between regenerative and gene transfer properties, indicating the possible significance of gene profiling results in parallel with the regenerative pathway events and their impact on plant cell receptivity to gene transfer.

## Conclusions

This paper presents the results of studies conducted at three observation levels (i.e., macroscopic, histological and molecular).

Surprisingly, to the best of our knowledge there has been no report to date that establishes the molecular interconnections between tissue culture and genetic transformation competencies in plant cells. Cells undergo an extensive change in gene regulation during the successive steps along a regenerative pathway. In this study, GST, MADS-box TF, SERK and RFc gene expression data provide spatio-temporal indicators relative to cell adjustment, chromatin remodeling, reprogramming of gene expression during SE induction and the proliferation state required for transgene integration, respectively.

Suitability for stable transformation is correlated with intense cell proliferation, as would be expected in any transformation procedure, but it also appears to be associated with indicators of a higher proportion of S phase-synchronized cells and a temporarily weakened defense system, as previously reported in the *Agrobacterium* transformation strategy.

We hypothesize that, in the course of the multi-step defense adaptation process that takes place during our in vitro culture protocol, the ideal moment for particle bombardment is probably an optimal period that coincides with (i) the chance to reach the nuclei of dividing cells (S-phase), (ii) the ease of passing through the physical barriers (before mature thickened walls are established in differentiating cells) and (iii) just before the establishment of durable biological defense mechanisms, one of whose functions would be to preserve genome integrity (between the first line of defense and the second wave of long-lasting defense processes).

As far as both tissue culture and direct gene transfer abilities are concerned, however, OXO activity appears to occur in parallel with the overall process of auxin-induced dedifferentiation, proliferation and differentiation, as well gene transfer competency over time. In the latter case, the involvement of OXO proteins, locally in cell wall structural reinforcement and globally in consolidating plant innate immunity, might be critical.

The idea that GerOXOs might be crucial during critical events in the life of plants was put forward some time ago [144, 145] and again more recently [149, 152, 186]. Such a broad and vital role in relaying stress response, adaptive behavior and cell survival might originate from the evolutionary history of this family of genes within the larger group of the multifunctional Cupin superfamily, of which the cereal OXOs are the archetypal members (for review, see [21, 31, 32]).

## Electronic supplementary material

Below is the link to the electronic supplementary material.

Supplementary material 1 (DOC 23 kb)
