# Supplementary material for: Analysis of expression profiles of selected genes associated with the regenerative property and the receptivity to gene transfer during somatic embryogenesis in Triticum aestivum L
Source: Mol Biol Rep. 2013 Sep 29;40(10):5883–906. doi: 10.1007/s11033-013-2696-y (PMC3825128; doi:10.1007/s11033-013-2696-y)
Supplement: Supplementary file 1 — Supplementary material 1 (DOC 23 kb) [file 11033_2013_2696_MOESM1_ESM.doc]

# Additional files

### Additional file 1 – Partial sequence of wheat cDNA encoding a MADS-domain transcription factor

We cloned from wheat a putative homologue to the maize *ZmMADS1* transcription factor [GenBank:AF112148]. The latter is a MIKC-type MADS-box family member specifically induced in maize embryogenic cultures .

**CCTTCTCCAAGCGCCGCAACGGCCTGCTCAAGAAGGCCTACGAGCTCTCCGTGCTCTGCGACGCCGAGGTGGCGCTCATCATCTTCTCCAGCCGCGGCAAGCTCTACGAGTTC 113 bp**
